# Biofertilizer effect of some zinc dissolving bacteria free and encapsulated on *Zea mays* growth

**DOI:** 10.1007/s00203-023-03537-5

**Published:** 2023-04-21

**Authors:** Asmaa Ahmed Yassen Ahmed Sultan, Hassan Mahmoud Gebreel, HebatAllah Ibrahim AbdElazeim Youssef

**Affiliations:** Department of Microbiology, Faculty of Science, Ain Shams University, El-Khalyfa El-Mamoun Street Abbasya, Cairo, Egypt

**Keywords:** Zinc-solubilizing bacteria, Biofertilizer, Sodium alginate *Acinetobacter*, *Bacillus*, *Stenotrophomonas*

## Abstract

Crop nutrition depends on zinc for enzymatic, oxidative, and metabolic processes. In the current study 20 different bacteria were isolated from five soil samples collected from different fields in Egypt. Bacterial isolates were screened for their ability to solubilize insoluble zinc oxide and zinc carbonate. The ability of selected isolates to tolerate soluble zinc was determined using different concentrations of (ZnSO_4_). Three bacterial isolates were selected with efficiency in solubilizing zinc oxide and zinc carbonate while tolerating high levels of soluble zinc. Molecular identification by 16S rRNA sequencing of the chosen isolates identified them as B3 (*Acinetobacter calcoaceticus*), B5 (*Bacillus proteolyticus*) and C6 (*Stenotrophomonas pavanii*). Sodium alginate beads formulated with the isolated bacteria were tested for stability under different storage conditions for 3 months. A pot experiment was conducted to study and compare the effect of using chosen isolates as an in vivo Zn solubilizer with amended ZnCO_3_ either alone or embedded in beads as carrier in the soil and its effect on growth parameters of *Zea mays* after 2 months. There was an increase in Zn uptake in all treatments compared to the control. However, plants grown in a pot treated with ZnCO_3_ and *Acinetobacter calcoaceticus* showed the highest zinc content and plant dry weight as compared to the control. Finally, selected isolates in both free and encapsulated forms showed improved plant growth parameters and higher zinc content and can be applied as biofertilizers to enhance soil fertility.

**Supplementary Information:**

The online version contains supplementary material available at 10.1007/s00203-023-03537-5.

## Introduction

Soil zinc deficiency affects millions of cropland worldwide, and is particularly prevalent in developing countries. In plants, Zinc is involved in carbohydrate metabolism (Alloway 2008). Zinc is imperative for both human development and crop production. Egypt's soils are among those that tend to be deficient in zinc (Khafagy et al. 2017), soils in areas where wheat is grown often have extremely low levels of plant-available P and Zn, causing widespread P and zinc deficiency in this crop (Kotb 2007). Zn deficiency can affect the development of plants and animals since Zn is a regulatory co-factor and structural constituent in proteins and enzymes involved in many biochemical pathways (Cakmak et al. 2017). In addition to carbohydrate metabolism, photosynthesis, and glucose metabolism, these enzymes are implicated in starch and sugar metabolism. In addition, Zn is also vital for protein metabolism, auxin metabolism, pollen formation, maintenance of the integrity of biological membranes and those related to resistant to pathogens (Rashid 1996). Zn acts as a significant antioxidant. The lack of zinc in plants can lead to retarded shoot growth, chlorosis, reduced leaf size, susceptibility to heat, light and fungal infections, as well as affecting grain yield, pollen formation, root development, and water uptake (Tavallali et al. 2010). Almost half of the world's cereal crops are grown on zinc-deficient soils; as a result, zinc deficiency in humans is a widespread problem. Soil Zn content is determined by the geochemical composition and weathering of the parent rock. Insoluble Zn cannot be assimilated by crops, resulting in Zn deficiency; more Zn fertilizers are applied to crops to combat Zn deficiency. However, this technique is costly and can be harmful to both human health and the natural environment. Thus, eco-friendly and cost-effective agro-technologies are needed to increase crop yield and reduce Zn deficiency (Khanghahi et al. 2021). When the concentration of Zn available in soil becomes less than 0.5 mg Zn/kg dry soil, deficiency symptoms become visible (Alloway 2001). Despite most soils having a total Zn content of around 50–100 mg l^−1^, soil solutions are usually extremely low in Zn concentration (0.002–0.196 mg l^−1^). (Srivastava and Gupta 1996). Fertilization appears a quick solution to rectify the nutrient deficiency but the cost of micronutrient fertilizers is high. The continuous use of inorganic fertilizers may cause damage to the physical, chemical and biological properties of soil, which could lead to the decline of soil fertility. Various methods have been used including zinc sulfate (White and Broadly 2005) or Zn-EDTA (Karak et al. 2005) as zinc fertilizers. However, their usage is not economical feasible or environment friendly and they are transformed into insoluble complex forms within 7 days of fertilizer application (Rattan and Shukla 1991). The use of plant growth-promoting microorganisms is a novel approach in this respect (Alavi et al. 2008). As bacteria release chelating metabolites in the rhizosphere of plants, siderophores are considered important reserves of micronutrients, like Zn and Fe that are easily available to plants. (Ahemad and Kibert 2014). Microbial siderophores make complexes with Zn and increases plant uptake (Madsen et al. 2012). There have been previous reports on transforming insoluble Zn into plant-accessible, soluble forms by soil microbiome. Bacterial genera, such as *Pseudomonas, Bacillus, Acinetobacter, Azotobacter, Azospirillum, Gluconacetobacter, Burkholderia*, and *Thiobacillus* have shown their ability to solubilize Zn (Bhakat et al. 2021). ZSB have the ability to improve crop quality via producing various phytohormones and soluble nutrients (e.g., P and K), synthesizing exopolysaccharides and siderophores, and reducing environmental stresses (Gupta et al. 2022). Using a pot experiment as a model, this study investigated the selection of powerful Zn solubilizing bacteria that could infiltrate edible parts of the crop and improve its Zn accumulation. The chosen bacteria will also be formulated in sodium alginate beads and their viability will be tested over 3 months during storage.

## Materials and methods

### Sample collection and isolation of zinc solubilizing bacteria on solid media

Five soil samples were collected from different fields (El Monofia and Giza) in Egypt. Twenty bacterial isolates of different morphology were selected after using proper dilutions from each soil sample through serial dilution on modified Bunt and Rovira medium containing gl^−1^ [0.4 KH_2_PO_4_, 0.5 (NH_4_)_2_SO_4_, 0.5 MgSO_4_·7H_2_O, 0.1 MgCl_2_, 0.1 FeCl_3_, 0.1 CaCl_2_, 1.0 peptone, 1.0 yeast extract, 5.0 glucose, 250.0 ml soil extracts, 20.0 g agar, 750.0 ml tap water, pH 7.0], as well as 0.1% insoluble ZnO and ZnCO_3_ as described by Saravanan et al. (2003). Bacterial isolates with high growth and clear halo zone were selected, purified, and preserved in 40% glycerol at −20 ℃ (Omara et al. 2016).

### Determination of zinc solubilization activity by isolated bacteria

The zinc-solubilizing potential of twenty selected bacteria was evaluated using two insoluble zinc sources: zinc oxide (ZnO), zinc carbonate (ZnCO_3_), and a combination of the two. The bacterial isolates were spotted on media in triplicate and incubated at 28 ℃ for 5 days to check for clear halo zones. By measuring the colony and halo zone diameter, hydrolysis capacity (HC) was calculated as follows: diameter of clear zone/diameter of colony (Omara et al. 2016).

### Determination of zinc tolerance by zinc-solubilizing bacterial isolates

At low concentrations, zinc is a nutrient, but at high concentrations, it is toxic. The ability of selected bacterial isolates to tolerate solubilized zinc was determined under in vitro conditions in nutrient broth containing different concentrations of soluble zinc (ZnSO_4_). The nutrient broth was prepared and ZnSO_4_ was integrated into the broth in such a manner that the final concentration of zinc was (20, 40, 50, 60, 80, 100, 150, 200, 300, 400 and 500) mg kg^−1^. These solutions were divided in 10 ml quantities in test tubes, sterilized and inoculated with 0.1 ml of each of the tested isolates. An un-inoculated control was also maintained. The total zinc-solubilizing bacterial population was assessed by plating on nutrient agar medium. The highest concentration at which poor growth was observed was taken as a tolerance level (Nandal and Solanki 2017).

### Molecular characterization of bacteria

The most efficient and tolerant isolates were chosen for molecular identification at Sigma Scientific Services Co., Giza, Egypt. DNA of the test bacterial isolates grown in nutrient broth was extracted with GeneJet Bacterial Genomic DNA Extraction Kit (Fermentas). The 16S rRNA gene of the isolate was amplified using universal primers forward and reverse (F, 5-AGA GTT TGA TCC TGG CTC AG-3 and R, 5-GGT TAC CTT GTT ACG ACT T-3) used to obtain a PCR product of ∼1.5 kb. The sample was placed in a hybrid thermal reactor thermocycler (Maxima Hot Start PCR Master Mix (Fermentas), initially denatured (enzyme activation) for 10 min at 95 ℃ for one cycle and denaturated for 30 s at 95 ℃, annealing for 1 min at 65 ℃ then extension for 1 min at 72 ℃. This was followed by a final elongation step for 10 min at 72 ℃. The PCR products were analyzed on 1% (*w/v*) agarose gels and sent to GATC (Germany) for sequencing by ABI 3730xl DNA sequencer. Sequence data were imported into the BioEdit version 5.0.9 sequence editor; base-calling was examined, and a contiguous sequence was obtained. Sequences used in the phylogenetic analysis were obtained from the RDP and GenBank databases. A phylogenetic tree was constructed using the neighbour-joining method (Omara et al. 2016).

### Storage experiment

#### Preparation of inoculums

A loop full of each tested zinc-solubilizing isolate on nutrient agar slant was individually inoculated in 250 ml nutrient broth medium in 500 Erlenmeyer flask at 28 ℃ in a shaking incubator for 24 h at 150 rpm.

#### Formulation of selected zinc-solubilizing bacteria with Alginate beads preparation

Alginate was prepared according to Bashan (1998) and Draget et al. (1997). Sodium alginate solution (6% *w/v*) were autoclaved at 121 ℃ for 20 min, then cooled, and mixed slowly with growth of each strain at the rate of (1:1 *v*/*v*). The alginate-cells mixture was stirred gently for 30 min at 100 rpm to be homogenous. For preparing the beads, the mixture was added drop wise, with the aid of a micropipette, to 0.1 M CaCl_2_ solution. The CaCl_2_ was then removed from the solution and the beads were washed twice with distilled sterile water. The beads were stored on sterile plates at room temperature and in the fridge. Viability of bacteria was tested every month for 3 months where 1 g alginate beads samples were rehydrated with NaCl solution (0.8% *W/V*, pH 7) while shaking based on Ivanova et al. (2005) then serial dilution of rehydrated bead sample and plate counting method was used for 10^7^ dilution of each (Mohamed et al. 2016).

### Plant experiment

#### Pot experiment to test the effect of biofertlizer on plant growth

Using sterile soil, the experiment was conducted in the greenhouse of the Microbiology department at Ain Shams University. The temperature during the experimental period (September and October) ranged from 30 ℃ to 37 ℃ with 10–12 h daylight and 60–65% relative humidity. According to the experiment plan, eight treatments referred to in (Table 1). Experiment was conducted in 18 cm pots filled with 2 kg of soil inoculated with 3 ml of each bacterial inoculant (B3, B5 and C6) and 120 beads. Each bead was 25 mm (B3 beads, B5 beads and C6 beads). The experiment was done in duplicate and arranged in a random pattern. Grain of maize (*Zea mays*) surface sterilization was performed in a 3.5% (*w/v*) solution of calcium hypochlorite for 10 min then submerged in distilled water twice, and afterwards each pot was seeded with one grain (Javed et al. 2018). Soil sample was sent to the research laboratories complex in Cairo University, Egypt for physical and chemical analysis of soil (Vaid et al. 2014). After 2 months, plants were harvested and dried in an electric oven at 70 ℃ for 72 h and dry weight **was** measured.

**Table 1:** Treatment design for pot experiment

Name	Treatment
T1	Negative control (no Zn source, no *bacteria*)
T2	Positive control (Zn source [ZnCO_3_], no *bacteria*)
T3	B3 (*Acinetobacter calcoaceticus*) and [ZnCO_3_]
T4	B5 (*Bacillus proteolyticus*) and [ZnCO_3_]
T5	C6 (*Stenotrophomonas pavanii*) and [ZnCO_3_]
T6	B3 (*Acinetobacter calcoaceticus*) in alginate beads and [ZnCO_3_]
T7	B5 (*Bacillus proteolyticus*) in alginate beads and [ZnCO_3_]
T8	C6 (*Stenotrophomonas pavanii*) in alginate beads and [ZnCO_3_]

#### Zinc content assay of plant materials

Samples were sent to Research laboratories complex in Cairo University, Egypt, for Zn content analysis using atomic absorption technique (Thermo Scientific iCE 3300, German) according to Christian and Feldman (1970).

## Results

### Isolation and screening of zinc-solubilizing bacteria on solid medium

**Fig. 1:**
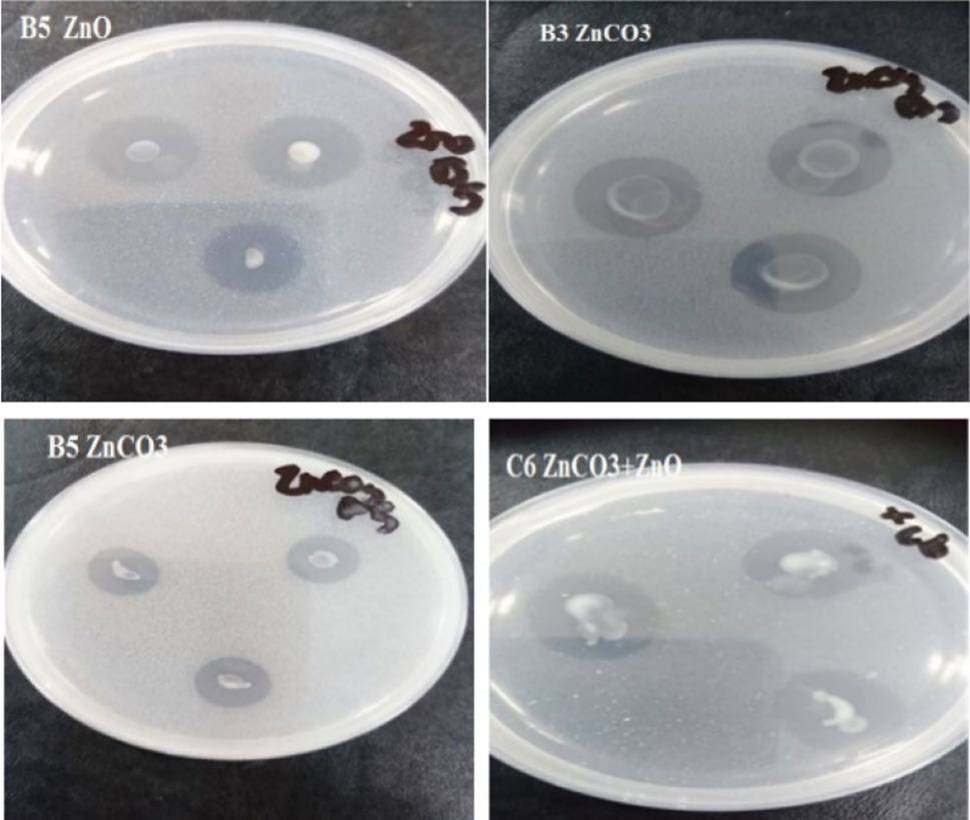
Clear zone by isolates on the modified Bunt and Rovira media agar plates Determination of zinc solubilization activity by isolated bacteria

20 isolates obtained and purified from soil samples were screened for hydrolysis capacity (HC) according to the diameter of the clear zone and of the colony on modified Bunt and Rovira solid medium containing insoluble ZnO, ZnCO_3_ and a combination of both. The results showed that most isolates were able to solubilize zinc to some degree but the most potent Zn-solubilizing isolates were A1, A5, B1, B3, B5, C1, C6 and D4, so they were selected for further studies (Fig. 1).

### Determination of zinc solubilization activity by isolated bacteria

**Fig. 2:**
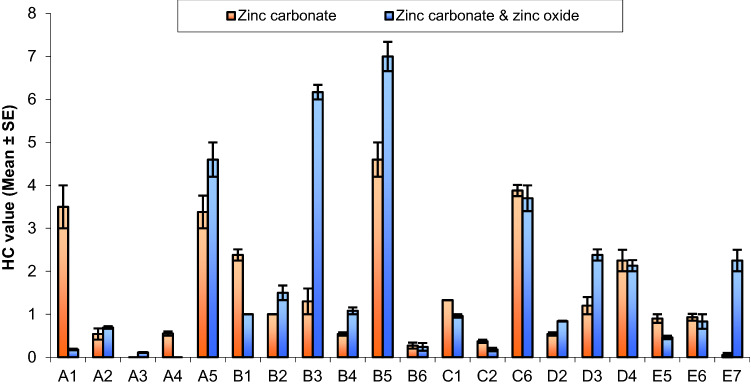
Hydrolysis capacity of bacterial isolates with zinc carbonate, zinc oxide and combination of both

Twenty isolates were screened according to hydrolysis capacity (HC) using the diameter of clear zone and diameter of colony on the modified Bunt and Rovira solid medium containing nonsoluble ZnO, ZnCO_3_ and combination of both as insoluble Zn source to choose the most effective bacteria for solubilizing Zn (Fig. 2 and Tables 2, 3, 4). The maximum hydrolysis capacity (HC: 7.00 ± 0.34) was observed in the bacterial isolates B5 supplemented with combination of both ZnO and ZnCO_3_ as insoluble Zn source followed by B3 bacterial isolates similarly having hydrolysis capacity (HC: 6.17 ± 0.17) supplemented with combination of both ZnO and ZnCO_3_ as insoluble Zn source. Also, B3 isolate showed the highest hydrolysis capacity of ZnO (HC: 5.50 ± 0.17) as insoluble Zn source. Moreover, C6 isolate had high hydrolysis capacity (HC: 5.13 ± 0.13) of ZnO, B5 isolate also had the highest hydrolysis capacity (HC: 4.60 ± 0.40) of isolates supplemented with ZnCO_3_ as Insoluble Zn source, followed by A5 and C6 isolates with hydrolysis capacity. Eight bacterial isolates (A1, A5, B1, B3, B5, C1, C6, and D4) showed the greatest potential and were selected for further studies.

**Table 2:** Solubilization capacity of zinc oxide by isolates under study

Code of bacterial isolate	Colony diameter (cm)	Clear zone diameter (cm)	HC value	Code of bacterial isolate	Colony diameter (cm)	Clear zone diameter (cm)	HC value
	Mean ± SE	Mean ± SE	Mean ± SE	Mean ± SE	Mean ± SE	Mean ± SE	Mean ± SE
A1	0.30 ± 0.00	0.75 ± 0.45	4.17 ± 0.17	B6	0.35 ± 0.05	1.50 ± 0.10	4.33 ± 0.33
A2	0.95 ± 0.05	0.70 ± 0.30	2.13 ± 0.13	C1	0.50 ± 0.00	0.85 ± 0.05	1.70 ± 0.10
A3	0.00 ± 0.00	0.00 ± 0.00	0.00 ± 0.00	C2	0.45 ± 0.05	0.10 ± 0.00	0.23 ± 0.03
A4	0.50 ± 0.00	0.70 ± 0.20	1.70 ± 0.10	C6	0.40 ± 0.00	2.05 ± 0.05	5.13 ± 0.13
A5	0.40 ± 0.00	0.95 ± 0.55	3.88 ± 0.13	D2	0.50 ± 0.10	0.10 ± 0.00	0.21 ± 0.04
B1	0.38 ± 0.03	0.95 ± 0.55	4.14 ± 0.14	D3	0.00 ± 0.00	0.00 ± 0.00	0.00 ± 0.00
B2	0.55 ± 0.05	0.25 ± 0.25	0.00 ± 0.00	D4	0.45 ± 0.05	0.75 ± 0.05	1.68 ± 0.08
B3	0.30 ± 0.00	0.95 ± 0.65	5.50 ± 0.17	E5	0.35 ± 0.05	0.20 ± 0.00	0.58 ± 0.08
B4	0.25 ± 0.05	0.40 ± 0.20	2.25 ± 0.25	E6	0.70 ± 0.10	0.40 ± 0.00	0.58 ± 0.08
B5	0.50 ± 0.00	1.10 ± 0.60	3.50 ± 0.10	E7	0.50 ± 0.00	0.85 ± 0.05	1.70 ± 0.10

Hydrolysis capacity (HC): diameter of clear zone/diameter of colony

**Table 3:** Solubilization capacity of zinc carbonate by isolates under study

Code of bacterial isolate	Colony diameter (cm)	Clear zone diameter (cm)	HC value	Code of bacterial isolate	Colony diameter (cm)	Clear zone diameter (cm)	HC value
	Mean ± SE	Mean ± SE	Mean ± SE	Mean ± SE	Mean ± SE	Mean ± SE	Mean ± SE
A1	0.25 ± 0.05	0.85 ± 0.05	3.50 ± 0.50	B6	0.35 ± 0.05	0.10 ± 0.00	0.27 ± 0.07
A2	1.25 ± 0.05	0.75 ± 0.05	0.54 ± 0.13	C1	0.90 ± 0.00	1.20 ± 0.00	1.33 ± 0.00
A3	1.25 ± 0.15	0.00 ± 0.00	0.00 ± 0.00	C2	0.55 ± 0.05	0.20 ± 0.00	0.37 ± 0.04
A4	0.55 ± 0.05	0.30 ± 0.00	0.55 ± 0.05	C6	0.40 ± 0.00	1.55 ± 0.05	3.88 ± 0.13
A5	0.40 ± 0.00	1.50 ± 0.00	3.38 ± 0.38	D2	0.75 ± 0.05	0.40 ± 0.00	0.54 ± 0.04
B1	0.40 ± 0.00	0.95 ± 0.05	2.38 ± 0.13	D3	0.80 ± 0.00	0.85 ± 0.05	1.20 ± 0.20
B2	0.50 ± 0.00	0.50 ± 0.00	1.00 ± 0.00	D4	0.45 ± 0.05	1.00 ± 0.00	2.25 ± 0.25
B3	0.95 ± 0.05	1.70 ± 0.10	1.30 ± 0.30	E5	0.45 ± 0.05	0.40 ± 0.00	0.90 ± 0.10
B4	0.65 ± 0.05	0.35 ± 0.05	0.54 ± 0.04	E6	0.60 ± 0.10	0.55 ± 0.05	0.93 ± 0.08
B5	0.45 ± 0.05	2.05 ± 0.05	4.60 ± 0.40	E7	0.50 ± 0.00	0.03 ± 0.03	0.05 ± 0.05

Hydrolysis capacity (HC): diameter of clear zone/diameter of colony

**Table 4:** Zinc carbonate and zinc oxide solubilization capacity of isolates

Bacterial isolate	Colony diameter (cm)	Clear zone diameter (cm)	HC value	Code of bacterial isolate	Colony diameter (cm)	Clear zone diameter (cm)	HC value
A1	0.55 ± 0.05	0.10 ± 0.00	0.18 ± 0.02	B6	0.20 ± 0.10	0.10 ± 0.00	0.24 ± 0.09
A2	1.15 ± 0.05	0.80 ± 0.00	0.69 ± 0.03	C1	1.20 ± 0.00	1.15 ± 0.05	0.96 ± 0.04
A3	0.95 ± 0.05	0.10 ± 0.00	0.11 ± 0.01	C2	0.70 ± 0.00	0.15 ± 0.05	0.18 ± 0.04
A4	0.80 ± 0.10	0.00 ± 0.00	0.00 ± 0.00	C6	0.65 ± 0.05	2.40 ± 0.00	3.70 ± 0.30
A5	0.45 ± 0.05	2.05 ± 0.05	4.60 ± 0.40	D2	0.65 ± 0.05	0.55 ± 0.05	0.84 ± 0.01
B1	0.15 ± 0.05	0.15 ± 0.05	1.00 ± 0.00	D3	0.40 ± 0.00	0.95 ± 0.05	2.38 ± 0.13
B2	0.30 ± 0.00	0.45 ± 0.05	1.50 ± 0.17	D4	0.40 ± 0.00	0.95 ± 0.05	2.13 ± 0.13
B3	0.30 ± 0.00	1.85 ± 0.05	6.17 ± 0.17	E5	0.65 ± 0.05	0.30 ± 0.00	0.46 ± 0.04
B4	0.65 ± 0.05	0.70 ± 0.00	1.08 ± 0.08	E6	0.55 ± 0.05	0.45 ± 0.05	0.83 ± 0.17
B5	0.30 ± 0.00	2.10 ± 0.10	7.00 ± 0.34	E7	0.45 ± 0.05	1.00 ± 0.00	2.25 ± 0.25

Hydrolysis capacity (HC): diameter of clear zone/diameter of colony

### Determination of Zn tolerance for the selected isolates

The solubilization of zinc might limit the growth of bacteria at higher levels. In vitro testing was performed on selected isolates using nutrient broth containing different concentrations of soluble zinc (ZnSO_4_) in order to determine their ability to tolerate solubilized zinc. The results in Table 5 showed that most of the isolates were tolerant isolates as they were able to grow and tolerate up to 400 mg kg^−1^ of ZnSO_4_ where only B3, B5 and C6 were able to grow at 500 mg kg^−1^ of ZnSO_4_.

**Table 5:** Zinc tolerance ability of the bacterial isolates

Conc. of Zinc	Name of isolates							
	A1	A5	B1	B3	B5	C1	C6	D4
20 mg kg^−1^	+	+	+	+	+	+	+	+
40 mg kg^−1^	+	+	+	+	+	+	+	+
50 mg kg^−1^	+	+	+	+	+	+	+	+
60 mg kg^−1^	+	+	+	+	+	+	+	+
80 mg kg^−1^	+	+	+	+	+	+	+	+
100 mg kg^−1^	+	+	+	+	+	+	+	+
150 mg kg^−1^	+	+	+	+	+	+	+	+
200 mg kg^−1^	+	+	+	+	+	+	+	+
300 mg kg^−1^	+	+	+	+	+	+	+	+
400 mg kg^−1^	−	+	+	+	+	+	+	+
500 mg kg^−1^	−	−	−	+	+	−	+	−

### Characterization of isolates

**Fig. 3:**
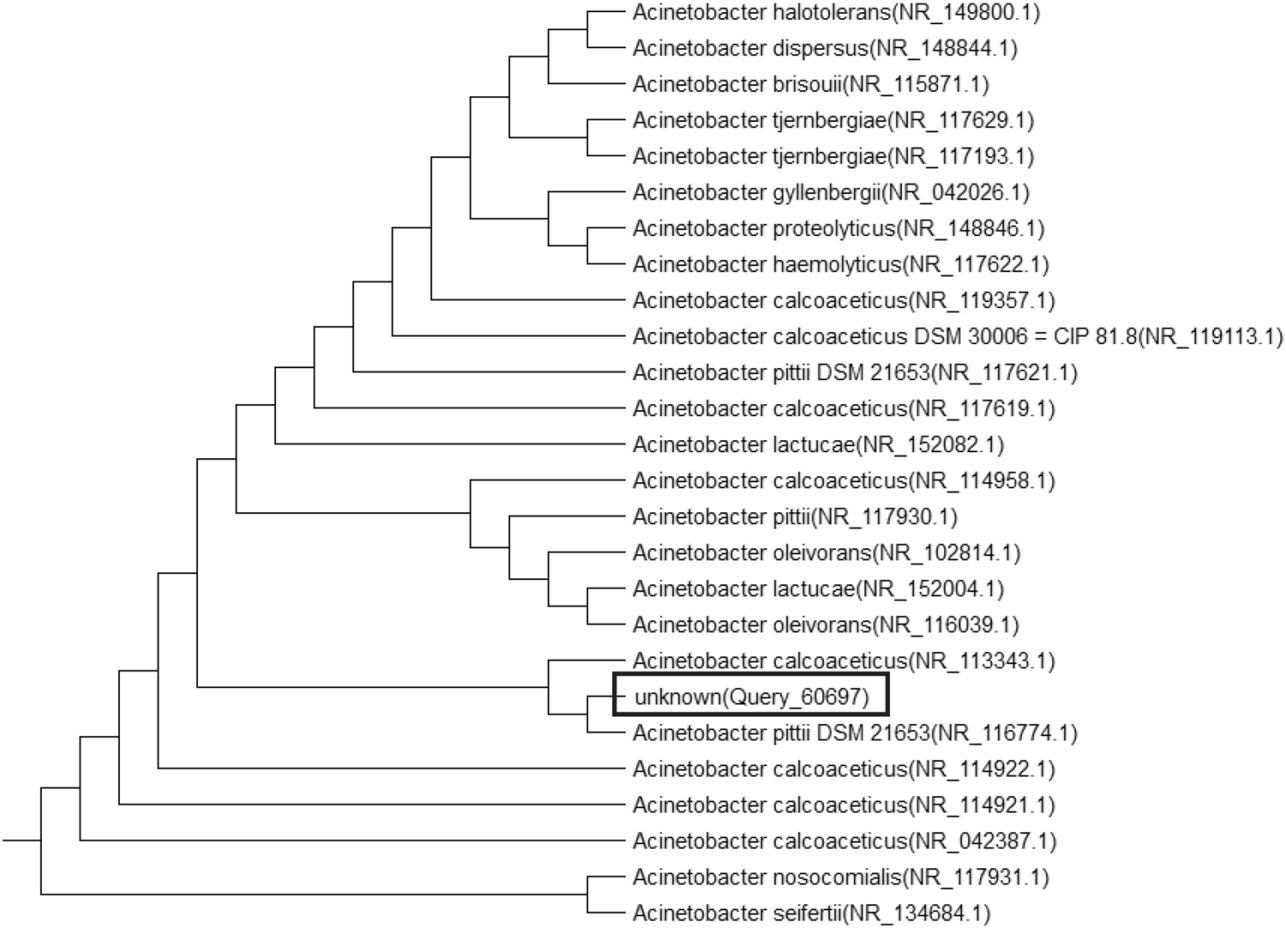
Neighbor-joining phylogenetic tree reconstructed on the basis of 16S rRNA gene sequence showing the phylogenetic *Acinetobacter calcoaceticus*

**Fig. 4:**
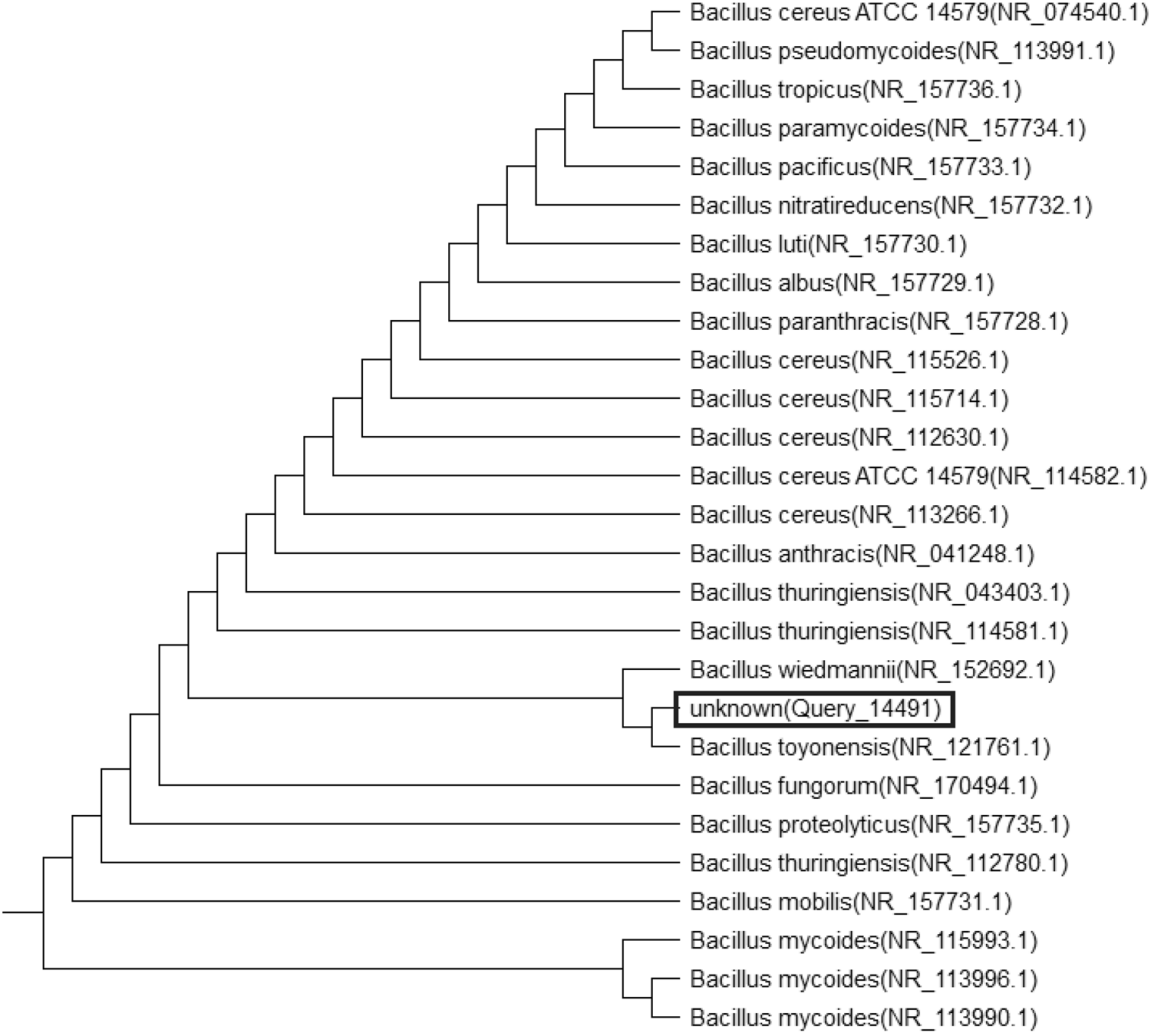
Neighbor-joining phylogenetic tree reconstructed on the basis of 16S rRNA gene sequence showing the phylogenetic *Bacillus proteolyticus*

**Fig. 5:**
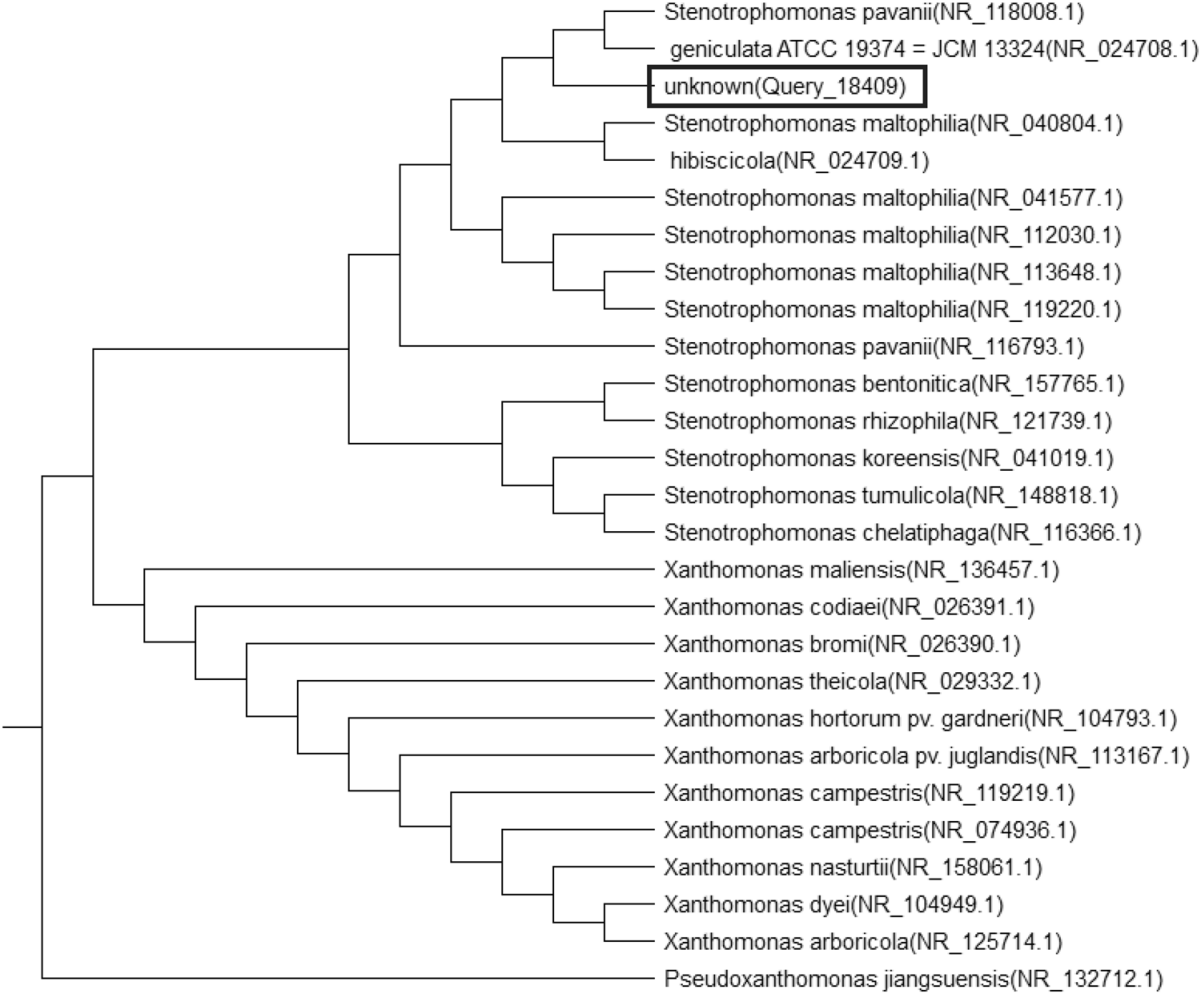
Neighbor-joining phylogenetic tree reconstructed on the basis of 16S rRNA gene sequence showing the phylogenetic *Stenotrophomonas pavanii*

Bacterial isolates designated as B3, B5 and C6 were isolated from mature compost. Colony morphology and Gram stain proved that: **B3** is white, flat, entire colonies with dry surface and Gram negative cocco-bacilli, **B5** is white, raised, entire colonies with smooth surface and Gram positive spore forming and **C6** is transparent, raised, entire colonies with glossy surface and Gram negative rod-shaped. The selected isolates were also characterized using 16S rRNA gene sequencing methods. Phylogenetic analysis based on 16S rRNA gene sequence was done by the Neighbor-joining method. Comparing obtained sequences with Gen bank database, isolates were found to have similarity with *Acinetobacter calcoaceticus* (with similarity, 100%), *Bacillus proteolyticus* (with similarity, 99.84%), *Stenotrophomonas pavanii*, (with similarity, 99.40%) respectively. Sequence data were deposited to GenBank with accession numbers in Table 6 and phylogenetic trees are shown in Figs. 3, 4 and 5.

**Table 6:** Molecular identification of selected isolates

Isolate	Identification	Accession numbers
B3	*Acinetobacter calcoaceticus*	NR 119,113.1
B5	*Bacillus proteolyticus*	NR 157,735.1
C6	*Stenotrophomonas pavanii*	NR 118,008.1

### Storage experiment

**Fig. 6:**
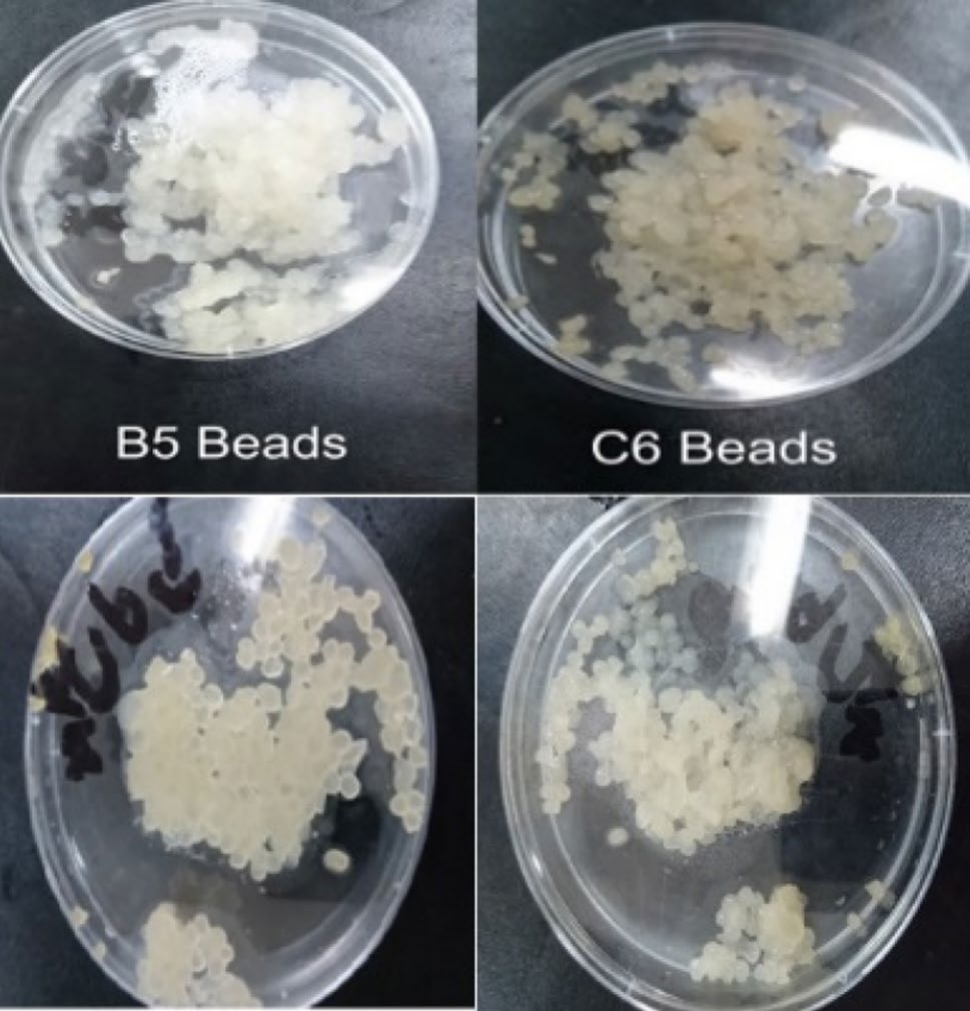
Encapsulation using alginate exerted high stability until the end of the storage period under Fridge and roomtemperatures

Since it is imperative the biofertilizer agent be viable during storage, the viability of the selected efficient isolates was determined with formulation in sodium alginate beads (Fig. 6) and at intervals during storage at room temperature and in cold storage for 3 months (Table 7). The results showed that the viable count of isolate B3 was 8.8 log_10_ CFU/ml at the start then was 8.8 log_10_ CFU/ml in the 1st month and remained 8.8 log_10_ CFU/ml after 3rd month of storage in room temperature also in fridge. It showed stable viability then decrease slightly (0.1 log 10 CFU/ml) after the 3rd month, For the number of B5 bacteria it started from 9.2 log_10_ CFU/ml and maintain the same count till the last month of storage in room temperature while, it increased by 0.1 log_10_ CFU/ml after the 3rd month in the fridge. Similarly, the number of C6 bacteria started from 8.9 log_10_ CFU/ml reached 9.15 log_10_ CFU/ml the 1st month. Then, it decreased to 8.9 log_10_ CFU/ml in the last month at room temperature while, it increased to 9.1 log_10_ CFU/ml after the 3rd month in the fridge.

**Table 7:** Viability of bacteria formulated in alginate beads during storage period

Sample	0 month (log_10_ CFU/ml)	1 month (log_10_ CFU/ml)	2 month (log_10_ CFU/ml)	3 month (log_10_ CFU/ml)
B3 R	8.82	8.89	8.85	8.87
B3 F		8.86	8.88	8.79
B5 R	9.27	9.29	9.27	9.28
B5 F		9.29	9.31	9.33
C6 R	8.96	9.15	9.10	8.99
C6 F		9.03	9.19	9.12

R room temperature (30 ℃)

F fridge (4 ℃)

### Effect of biofertilizers on plant growth

#### Pot experiment

**Fig. 7:**
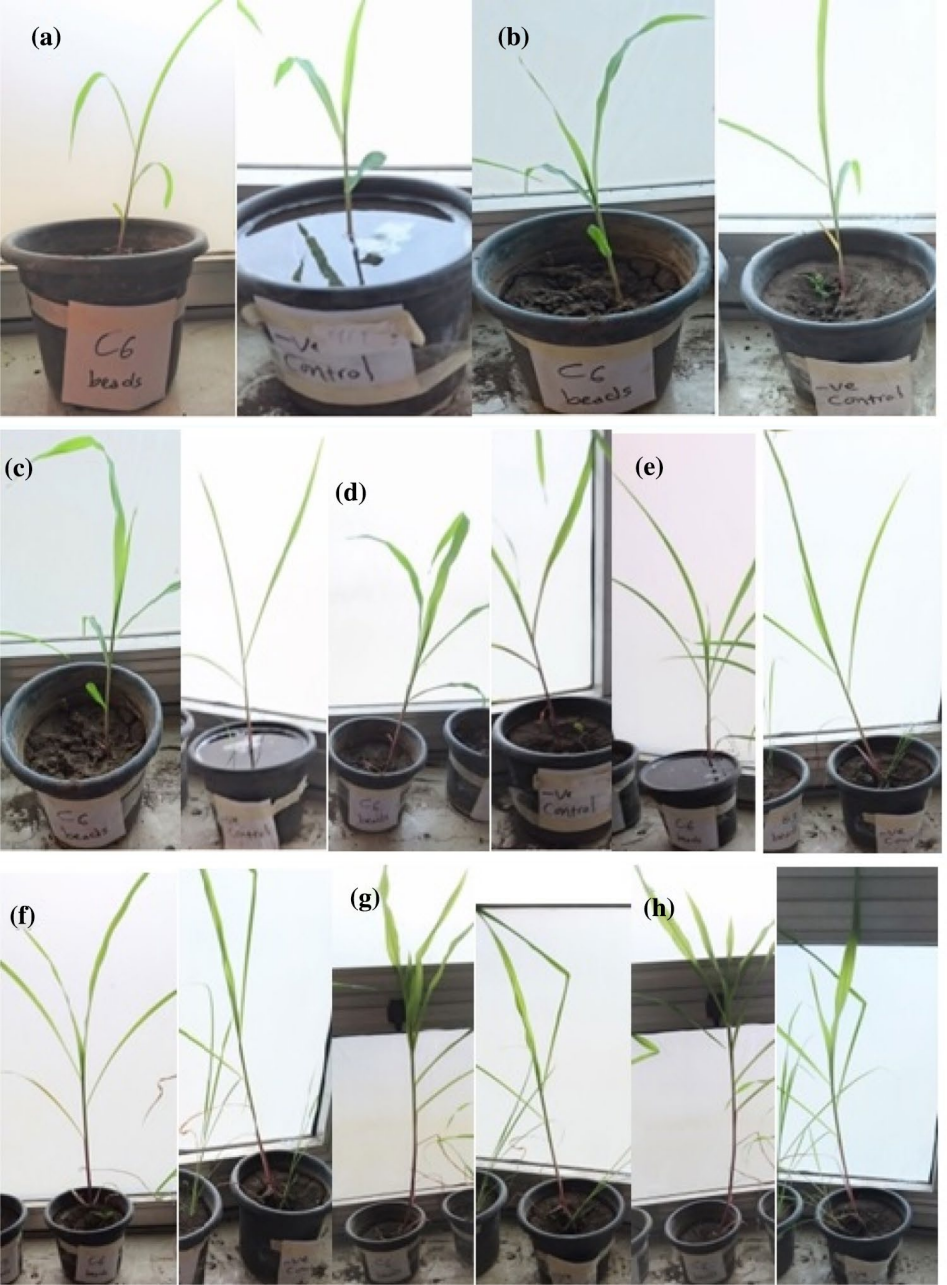
Pot experment soil inoculated with C6 beads compared to negative control during different growth stages **a** 1 week, **b** 2 weeks, **c** 3 weeks, d 4 weeks, **e** 5 weeks, **f** 6 weeks, **g** 7 weeks and h 8 weeks

Plant experiment was carried out using soil, which was analyzed to determine its physical and chemical characteristics (Table 8). Figure 7 showed the pots at different growth stages. The results showed considerable increase in shoot and root lengths, fresh and dry weights in all treatments over the negative control. This is because no zinc or bacteria was added to the sterile soil. As showed in (Table 9) the maximum fresh weight was 35.50 g for B3 followed by 32.25 gm for B5 beads and 31.95 gm for C6 beads, respectively. In addition, B3 had the best dry weight of 16.19 gm, followed by C6 beads 15.46 then (C6 and B3 beads). Furthermore, B3 had the highest shoot length of 32.20 cm, while, C6 30.75 cm and C6 beads was 30.35 cm. On the other hand, B5 had the highest root length 14.43 cm followed by C6 beads with 13.25 cm, and B5 beads with 12.75 cm.

**Table 8:** Analysis of physical and chemical properties of soil used for pot experiment

Property	Result
Texture	Clay loam
Organic carbon (%)	0.61
Sand (%)	27.03
Silt (%)	37.99
Clay (%)	34.99
pH (1:2) soil:water suspension	7.0
Fe (mg l^−1^)	0.336
Mn (mg l^−1^)	1.24
Zn (mg l^−1^)	0.343
Cu (mg l^−1^)	1.47

**Table 9:** Effect of biofertlizer inoculation on zinc content and dry weight in at 60 days

Treatment	Fresh weight	Dry weight	Shoot lengths	Root lengths	Zn content (mg kg^−1^)
	Mean ± SE	Mean ± SE	Mean ± SE	Mean ± SE	
(−ve) control	22.60 ± 1.60	12.09 ± 0.09	22.85 ± 0.85	9.05 ± 0.15	34.28
(+ve control)	25.90 ± 1.10	14.25 ± 0.76	25.45 ± 0.45	9.00 ± 0.10	109.24
B3	35.50 ± 0.50	16.19 ± 0.81	32.20 ± 0.80	12.70 ± 0.20	370.20
B5	29.65 ± 0.55	13.63 ± 0.53	29.15 ± 0.55	14.43 ± 0.27	200.60
C6	27.70 ± 0.30	14.77 ± 0.24	30.75 ± 0.75	11.00 ± 0.20	299.13
B3 beads	30.00 ± 0.30	14.72 ± 0.48	28.95 ± 0.15	11.75 ± 0.15	212.61
B5 beads	32.25 ± 0.75	13.72 ± 0.22	29.10 ± 0.60	12.75 ± 0.15	162.12
C6 beads	31.95 ± 1.95	15.46 ± 0.54	30.35 ± 0.35	13.25 ± 0.15	358.28

#### Zinc content assay of plant materials

**Fig. 8:**
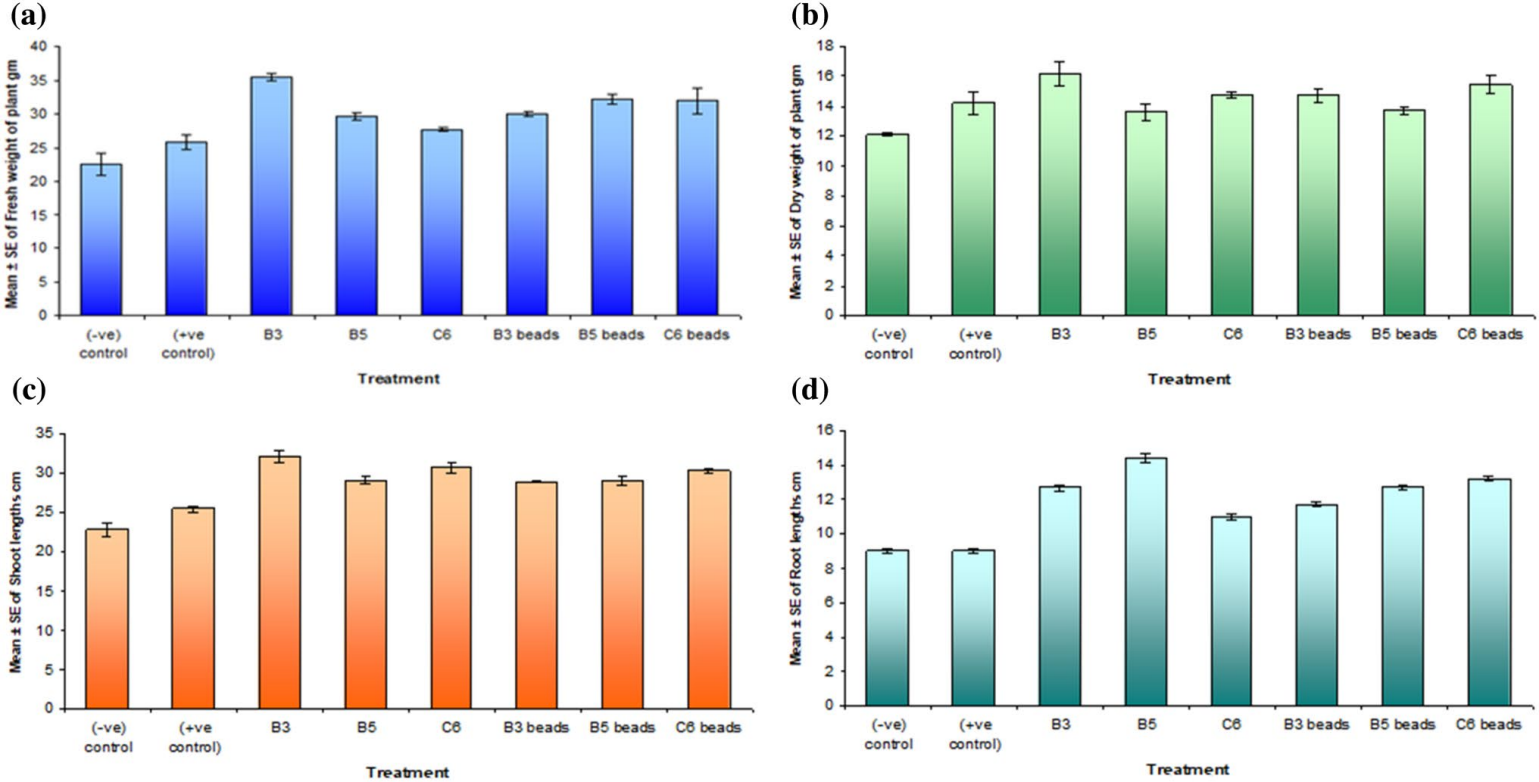
Effect of bacterial biofertlizers on growth parameters of plants harvested after 2 months. **A** Fresh weight of plant, **B** Dry weight of plant, **C** Shoot length, **D** Root length

The results showed that negative control plants where no zinc or bacteria was added to the soil had far lower zinc levels than positive control plants (Fig. 8). On the other hand, all treatments resulted in an increased zinc content of the analyzed plants. In addition, plants grown in the pot with free B3 isolate as a biofertilizer had the highest zinc content (370.20 mg kg^−1^) while plants with B3 in beads had only 212.61 mg kg^−1^; meanwhile, plants with free B5 inoculum showed higher zinc concentrations than those grown in beads formulated with the same isolate. The zinc content of beads loaded with isolate C6 was 358.28 (mg kg^−1^), while the zinc content of free C6 isolate inoculum was 299.13 (mg kg^−1^). Finally, it is clear that both selected isolates and isolates embedded in sodium alginate beads improved zinc content and growth of plants.

## Discussion

Globally, zinc deficiency in soil is a major problem because plants' Zn requirements are rarely met (Sillanpaa 1990)**.** There is an estimated 50% shortage of plant-available zinc in soils used for cereal production around the world (Graham and Welch 1996). Almost all crops and pastures worldwide suffer from zinc deficiency, leading to severe yield losses and nutritional deficiencies. This is particularly the case in areas of cereal crop (Alloway 2008)**.** Fertilizers are not always effective in correcting Zn deficiency due to economic and agronomic factors. There are several factors that contribute to reduced Zn availability in developing countries, including topsoil drying, soil constraints, disease interactions, and fertilizer costs. (Graham and Rengel 1993). Bacteria play a vital role in the processes of environmental cycling such as metal solubilization into soluble forms that are more suitable for uptake by plants. This study is focused on the isolation and identification of zinc dissolving bacterial strains and their ability to be used as biofertilizers based on their ability to solubilize zinc. Twenty zinc-solubilizing bacteria were isolated from soil collected from different farms in Egypt. Bhatt and Maheshwari, (2020) have previously isolated zinc solubilizing bacteria from cow dung using serial dilution protocols on Bunt and Rovira media containing 0.1% zinc sources, ZnO and ZnCO_3_, incubated for 1 week at 30° C. In contrast, Javed et al. (2018) stated that the plate method is limited and used both qualitative and quantitative methods to isolate zinc solubilizing bacteria. This study showed the ability of most isolates to tolerate soluble zinc in the form of (ZnSO_4_) up to 400 mg kg^−1^ only B3, B5 and C6 were able to survive 500 mg kg^−1^. Metal resistance mechanisms are highly dependent on metal interactions with cells, and the most likely cause of such high resistance is either bioaccumulation or biosorption. Atomic absorption spectroscopy studies revealed zinc bioaccumulation by zinc tolerant bacteria (ZTB). The ZTB strains were found to produce a significant amount of exopolysaccharide (EPS) under Zn stress. EPS mediated Zn biosorption mainly occurs due to the interaction between positively charged Zn ions and negatively charged EPS on the cell surfaces (Gupta and Diwan 2016). Which was supported by Pramanik et al. (2018) they isolated three bacterial strains and selection was based on their ability to grow in media containing 500 mg kg^−1^ of soluble zinc (ZnSO_4_). Similarly, Saravanan et al. (2003) results showed that both test isolates were able to survive 500 mg kg^−1^ but one was inhibited after 8 days incubation. On the contrary, Nandal and Solanki. (2017) showed that most of their isolates were only able to grow up to 100 ppm of ZnSO_4_ and only one isolate was able to tolerate 200 mg kg^−1^. Solubilization of Zn compounds by bacteria depends on organic acid production, especially 2-ketogluconic acid and H^+^, as well as other metabolic products, siderophores, and CO_2_ (Nautiyal et al. 2000). Sodium alginate was chosen as a carrier and tested for the ability to keep the biofertilizer agent viable during storage. Viability was compared during storage at both room temperate and fridge. Viable counts results showed that biofertlizer agents remained viable in both storage conditions during 3 months, where only B3 fridge sample showed slight decrease from 8.8 log_10_ CFU/ml to 8.7 log_10_ CFU/ml by the end of the storage period while all other samples showed stable counts. These results agreed with the results by Mohamed et al. (2016) who**,** recommended sodium alginate beads as best carrier as it is a cheap and easily used and stored. Encapsulation using alginate exerted high stability densities up to the end of 6 months storage period at 5, 20 and 30 ℃, likewise this study showed that sodium alginate beads are able to maintain viability of bacteria for 3 months in room temperature and fridge. The results agreed with those of Bashan (1998) who mentioned that bacteria could survive in alginate beads for long periods. Moreover, Ivanova et al. (2005) found that total number of bacteria decreased quickly in the first 7 days of storage, but total number of viable bacteria remained stable for 6 months of storage. Also, plant growth-promoting bacteria, *Bacillus subtilis* and *Pseudomonas corrugata*, immobilized in sodium alginate-based formulation were evaluated for their survival, viability and plant growth-promoting ability after 3 years of storage at 4 ℃ (Trivedi and Pandey 2008). Populations of both bacterial isolates recovered from the immobilized sodium alginate beads were in the order of 10^8^ cfu/g. The plant-based bioassay indicated that the plant growth promotion ability of both of the bacterial isolates was equal to those of fresh broth-based formulations. The bacterial isolates retained root colonization, and antifungal and enzyme activities in the alginate-based formulation during storage (Trivedi and Pandey 2008). In this study, three bacteria were selected based on their ability to solubilize zinc in different forms and their ability to tolerate high amounts of zinc and were identified as B3 (*Acinetobacter calcoaceticus*), B5 (*Bacillus proteolyticus*), C6 (*Stenotrophomonas pavanii*). Plant experiment was very helpful for analyzing the effects of microbial species on various plant growth parameters as well as on zinc content and evaluating their efficacy under simulatory field conditions when maize seeds were planted in soil inoculated with these strains, they showed enhanced growth as well as increase in zinc content over both negative control where no zinc or bacteria was added as well as positive control where no bacteria was added only ZnCo_3_ was added. The results varied with inoculation of bacterial isolate alone or as beads with ZnCo_3_. There were instances when inoculation of bacterial isolate showed better results than the addition of beads as with B3 as well as B5 with B3 isolate having highest zinc continent of 370.20 Zn (mg kg^−1^), while C6 isolate the beads had better results where encapsulated C6 had second highest zinc content 358.28 Zn (mg kg^−1^). Similarly, Goteti et al. (2013) demonstrated that inoculation with plant growth-promoting rhizobacteria significantly enhanced the growth of maize in all dimensions. While, Omara et al. (2016) indicated that inoculation with *E. cloacae* alone or with different zinc applications variably increased zinc percentage of *Zea mays* with the highest content of Zn in plants reported for inoculation with *E. cloacae* combined with ZnO. Similar approach by Zaheer et al. (2019) showed that *Pseudomona* ssp. strain AZ5 and *Bacillus* sp. strain AZ17 inoculation improved considerably the number and dry weight of nodules, grain, and straw weight, and uptake of P and Zn of the chickpea cultivar compared to non-inoculated control. A similar finding was made by Kamran et al. (2017) using Zinc solubilizing *Pseudomonas fragi, E. cloacae*, and *Rhizobium* sp. When they inoculated wheat plants with these strains, showed enhanced shoot and root length and weight as well as zinc content. While, Yasmin et al. (2021) findings showed that In the treatment with Zn-solubilizing bacteria *P. protegens* RY2 and ZnO as zinc source, zinc content in both root and shoot was significantly (P 0.05) higher than in the control treatment.. Plant growth and development are enhanced by zinc-solubilizing bacteria colonizing the rhizosphere and intensifying zinc bioavailability by solubilizing complex zinc compounds. Enhancement of the soil microbiome has the potential to increase crop production (Yuan et al. 2019), and reduce chemical inputs (Thijs et al. 2016), resulting in more sustainable agricultural practices. In conclusion, the current study findings represent contribution to achieve sustainable agriculture by introducing a cheap biofertilizers of three selected different bacteria formulated in stable commercial form. Sodium alginate encapsulated biofertilizers displayed viability at both room and fridge temperatures during storage and lasting for 3 months. And also, they showed enhanced zinc content and increased growth parameters of the plants during a pot experiment conducted in greenhouse. Hence, can be used for biofortification of *Zea mays*, which in turn improves human and animal health.

## Supplementary Information

Below is the link to the electronic supplementary material.

Supplementary file1 (TXT 1 KB)

Supplementary file2 (TXT 1 KB)

Supplementary file3 (TXT 1 KB)

Supplementary file4 (TXT 1 KB)

Supplementary file5 (TXT 2 KB)

Supplementary file6 (TXT 2 KB)

## References

[CR1] Ahemad M, Kibret M (2014). Mechanisms and applications of plant growth promoting rhizobacteria: current perspective. J King Saud Univ Sci.

[CR2] Alavi S, Bugusu B, Cramer G, Dary O, Lee TC, Martin L, McEntire J, Wailes E (2008). Rice fortification in developing countries: a critical review of the technical and economic feasibility.

[CR3] Alloway BJ (2001). Zinc the vital micronutrient for healthy, high-value crops.

[CR4] Alloway BJ (2008). Zinc in soils and plant nutrition.

[CR5] Bashan Y (1998). Inoculants of plant growth-promoting bacteria for use in agriculture. Biotechnol Adv.

[CR6] Bhakat K, Chakraborty A, Islam E (2021). Characterization of Zinc solubilization potential of arsenic tolerance *Burkho* Spp. isolated from rice rhizospheric soil. World J Microbiol Biotechnol.

[CR7] Bhatt K, Maheshwari DK (2020). Zinc solubilizing bacteria (*Bacillus megaterium*) with multifarious plant growth promoting activities alleviates growth in *Capsicum annuum* L. 3. Biotech.

[CR8] Cakmak I, McLaughlin M, White P (2017). Zinc for better crop production and human health. Plant Soil.

[CR9] Christian GD, Feldman FJ (1970). Atomic absorption spectroscopy applications in agriculture biology and medicine.

[CR10] Draget KI, Skjåk-Bræk G, Smidsrød O (1997). Alginate based new materials. Int J Biol Macromol.

[CR11] Goteti PK, Emmanuel LD, Desai S, Shaik MH (2013). Prospective Zinc solubilising bacteria for enhanced nutrient uptake and growth promotion in Maize (*Zea mays* L). Int J Microbiol.

[CR12] Graham RD, Welch RM (1996) Breeding for staple-food crops with high micronutrient density working papers on agricultural strategies for micronutrients. International Food Policy Research Institute: Washington DC

[CR13] Graham RD, Rengel Z, Robson D (1993). Genotypic variation in Zn uptake and utilization by plants. Zinc in soils and plants.

[CR14] Gupta P, Diwan B (2016). Bacterial exopolysaccharide mediated heavy metal removal: a review on biosynthesis, mechanism and remediation strategies. Biotechnol Rep.

[CR15] Gupta R, Kumari A, Sharma S, Alzahrani OM, Noureldeen A (2022). Identification, characterization and optimization of phosphate solubilizing rhizobacteria (PSRB) from Rice rhizosphere. Saudi J Biol Sci.

[CR16] Ivanova E, Teunou E, Poncelet D (2005). Encapsulation of water sensitive products: effectiveness and assessment of fluid bed dry coating. J Food Eng.

[CR17] Javed H, Akhtar MJ, Asghar HN, Jamil A (2018). Screening of Zinc solubilizing bacteria and their potential to increase grain concentration in wheat (*Triticumae stivum*). Inter J Agri Boil.

[CR18] Kamran S, Shahid I, Baig DN, Rizwan M, Malik KA, Mehnaz S (2017). Contribution of Zinc solubilizing bacteria in growth promotion and Zinc content of wheat. Front Microbiol.

[CR19] Karak T, Singh UK, Das S, Das DK, Kuzyakov Y (2005). Comparative efficacy of ZnSO_4_ and Zn-EDTA application for fertilization of rice (*Oryza sativa* L.). Arch Agron Soil Sci.

[CR20] Khafagy EEE, Mosaad ISM, Seadh AK (2017). Interaction effect between mineral zinc-nitrogen fertilization mixture and organic fertilization as compost on yield, nutrients uptake of rice and some soil properties. Agric Eng Int CIGR J.

[CR21] Khanghahi MY, Strafella S, Allegretta I, Crecchio C (2021). Isolation of bacteria with potential plant-promoting traits and optimization of their growth conditions. Curr Microbiol.

[CR22] Kotb MAA (2007). Alleviation the negative effects of phosphorus-zinc antagonism on growth and yield of wheat (*Triticumae stivum *L.) grown in newly reclaimed sandy soils. Product Dev.

[CR23] Madsen LH, Borg S, Brinch-Pedersen H, Tauris B, Darbani B, Noeparvar S, Holm PB (2012). Wheat ferritins: improving the iron content of the wheat grain. J Cereal Sci.

[CR24] Mohamed ASA, Khider A, Muniandy S (2016). Effect of Storage Temperature, Duration and Types of Biofertilizer Carriers on Survival and Numbers of Bacterial Strains Bacillus *megaterium* var. *phosphaticum* , *Azotobacter chroococcum*, *Rhizobium*
*leguminosarum* and Transformant, Transconjugant B. *megaterium* var. *phosphaticum*. International Conference on Agricultural Food Biological and Health Sciences.

[CR25] Nandal V, Solanki M (2017). Isolation and identification of Zinc dissolving bacteria and their potential on growth of *Zea mays*. Int J Basic Appl Biol.

[CR26] Nautiyal CS, Bhadauria S, Kumar P, Lal H, Mondal R, Verma D (2000). Stress induced phosphate solubilization in bacteria isolated from alkaline soils. FEMS Microbiol Lett.

[CR27] Omara AA, Ghazi AA, El-Akhdar IA (2016). Isolation and identification of Zinc dissolving bacteria and their potential on growth of *Zea mays*. Egypt J Microbiol.

[CR28] Pramanik K, Mitra S, Sarkar A, Maiti TK (2018). Alleviation of phytotoxic effects of cadmium on rice seedlings by cadmium resistant PGPR strain *Enterobacter aerogenes* MCC 3092. J Hazard Mater.

[CR29] Rashid A (1996) Nutrient indexing of Cotton in Multan district and Boron and Zinc nutrition of Cotton. Micronutrient Project Annual Report 1994–95 National Agricultural Research Center, Islamabad. p76

[CR30] Rattan RK, Shukla LM (1991). Influence of different zinc carriers on the utilization of micronutrients by rice. J Indian Soc Soil Sci.

[CR31] Saravanan VS, Subramanian R, Raj A (2003). Assessing in vitro solubilization potential of different zinc solubilizing bacterial (zsb) isolates. Brazilian. J Microbiol.

[CR32] Sillanpaa M (1990). Micronutrients assessment at the country level: an international study FAO Soils Bulletin 63.

[CR33] Srivastava PC, Gupta UC (1996). Trace elements in crop production.

[CR34] Tavallali V, Rahemi M, Eshghi S, Kholdebarin B, Ramezanian A (2010). Zinc alleviates salt stress and increases antioxidant enzyme activity in the leaves of pistachio (*Pistaciavera *L*. ‘Badami’*) seedlings. Turk J Agr Forest.

[CR35] Thijs S, Sillen W, Rineau F, Weyens N, Vangronsveld J (2016). Towards an enhanced understanding of plant-microbiome interactions to improve phytoremediation: engineering the metaorganism. Front Microbiol.

[CR36] Trivedi P, Pandey A (2008). Recovery of plant growth-promoting rhizobacteria from sodium alginate beads after 3 years following storage at 4 C. J Ind Microbiol Biotechnol.

[CR37] Vaid SK, Kumar B, Sharma A, Shukla AK, Srivastava PC (2014). Effect of zinc solubilizing bacteria on growth promotion and zinc nutrition of rice. J Soil Sci Plant Nutr.

[CR38] White PJ, Broadly MR (2005). Biofortifying crops with essential mineral elements. Trends Plant Sci.

[CR39] Yasmin R, Hussain S, Rasool MH, Siddique MH, Muzammil S (2021). Isolation, characterization of Zn solubilizing bacterium (Pseudomonas protegens RY2) and its contribution in growth of chickpea (*Cicerarietinum* L) as deciphered by improved growth parameters and Zn content. Dose Response.

[CR40] Yuan Y, Brunel C, van Kleunen M, Li J, Jin Z (2019). Salinity-induced changes in the rhizosphere microbiome improve salt tolerance of *hibiscus hamabo*. Plant Soil.

[CR41] Zaheer A, Malik A, Sher A, Mansoor Qaisrani M, Mehmood A, Ullah Khan S, Ashraf M, Mirza Z, Karim S, Rasool M (2019). Isolation, characterization, and effect of phosphate-zinc-solubilizing bacterial strains on chickpea (*Cicerarietinum *L.) growth. Saudi J Biol Sci.

